# Enhancing access and clinical triage in primary eye care through digital vision testing: validation of the SightConnect mobile application

**DOI:** 10.3389/fdgth.2026.1750207

**Published:** 2026-05-08

**Authors:** Payal Sangani, Chodup Thinley, Mohit Pushpwan, Rahul Negi, Mahesh Mandawar, Krishna Deshagiri, Niranjan Kumar Yenamala, Md Hasnat Ali, Pravin K Vaddavalli, Pinakin Gunvant Davey, Padmaja Kumari Rani

**Affiliations:** 1 College of Optometry, Western University of Health Sciences, Pomona, CA, United States; 2 Suven Clinical Research Center, LV Prasad Eye Institute, Hyderabad, India; 3 Bausch and Lomb School of Optometry, Brien Holden Institute of Optometry and Vision Sciences, LV Prasad Eye Institute, Hyderabad, India; 4 Department of Refractive Services, LV Prasad Eye Institute, Hyderabad, India; 5 Center for Technology Innovation, LV Prasad Eye Institute, Hyderabad, India; 6 Gullapalli Pratibha Rao International Centre for Advancement of Rural Eye Care, LV Prasad Eye Institute, Hyderabad, India; 7 Clinical Epidemiology and Biostatistics, LV Prasad Eye Institute, Kallam Anji Reddy Campus, Hyderabad, India; 8 Department of Cataract and Refractive Surgery, Shantilal Shanghvi Cornea Institute, LV Prasad Eye Institute, Hyderabad, India; 9 Anant Bajaj Retina Institute, LV Prasad Eye Institute, Hyderabad, India

**Keywords:** digital health, digital vision chart, refractive error, tele-ophthalmology, vision screening, vision test, visual acuity

## Abstract

**Background:**

Assessment of vision is the vital first step of an eye exam for determining visual status. The availability of portable digital vision testing tools enables vision assessments in remote areas, at home and during teleconsultations.

**Purpose:**

To assess the accuracy and agreement for measuring vision using the SightConnect mobile application (SightConnect) compared to in-clinic standard vision testing using the charts and to evaluate the performance of the SightConnect visual acuity grading algorithm, which classifies the visual acuity into one of these categories of visual impairment: mild, moderate and severe.

**Methods:**

Individuals visiting LV Prasad Eye Institute as part of their ophthalmic consultation were consecutively recruited for this study. Part -1 of the study evaluated the near visual acuity with SightConnect whereas part-2 evaluated both the distance and near acuity. The standard visual acuity at both distance and near was assessed by trained optometrists and vision technicians. SightConnect further classifies the obtained visual acuity based on severity of the vision and uses them as reference to suggest urgency of referral for an eye exam, as routine, early and urgent need for clinical attention.

**Results:**

A total of 1,481 individuals were assessed for visual acuity using SightConnect. Of these 710 participants in part-1 and 771 participants in part-2. Both eyes of the study participants were included in the study. The mean difference in near visual acuity assessment between SightConnect and the clinical measurements was 0.08 logMAR (−4 letters). For distance acuity, the mean difference between SightConnect and clinical measurements was 0.09 logMAR. The intraclass correlation coefficients indicated moderate reliability, with ICCs of 0.74 for near and 0.67 for distance acuity measurements. The visual acuity grading module was 78 to 90% accurate for identifying routine, early and urgent referrals based on vision status.

**Conclusions:**

Visual acuity testing using the SightConnect mobile application reported moderate agreement with the standard clinical assessments, and the accuracy in grading visual acuity and suggesting referral urgency was acceptable. These findings support the use of SightConnect applications for enabling and enhancing access to primary eye care services and guiding users on when to seek clinical attention.

## 1 Introduction

Visual impairment and blindness remain important global health issues of this century despite various advances in healthcare and ophthalmology. Twenty-five percent of the global population (∼2.2 billion individuals) are living with visual impairment symptoms (1). Most of the causes of visual impairment and blindness could be prevented with early detection and timely management. However, the availability and access to eye care services along with socio-economic status have remained a barrier for addressing and treating eye conditions in a timely fashion, especially in Low-Middle Income Countries (LMICs) (2).

The concept of telemedicine has been in existence for a long while all over the world (3, 4). Telemedicine thus far has been predominantly focused on taking medical care to rural places where patients do not have access to medical facilities either basic or advanced. Patient symptoms and measurement of visual acuity are the key parameters that eye care practitioners assess to evaluate and monitor the eye conditions. It helps clinicians in quantifying the present vision status and mostly as an indicator of severity of disease state. Testing visual acuity through a mobile application and using severity of vision status to determine need for an eye checkup can facilitate referral services and have potential to improve the quality of care in teleophthalmology.

Snellen type and ETDRS logMAR chart based visual acuity measurements are widely utilized in clinical settings (5). The ETDRS chart requires a large lightbox, electrical power and is not portable. It requires significant amount of space and testing distance. Additionally, the luminance of the chart should be calibrated to 85 cd/m^2^, which is obtained using a retro illuminated light box and requires special equipment for calibration. These situations pose difficulties in using them as telemedicine tools. To address these issues and ease of use in telemedicine, several digital software and mobile applications are developed that help test visual functions, visual acuity, color vision, Amsler visual fields. Some of the commonly used apps are PeekAcuity, WHOeyes, Smart Optometry, Eye Patient, etc (6). Such smartphone-based apps are easy to download and can effectively check a patient's distance and near visual acuity. With proper orientation and training, non-ophthalmic professionals can utilize these applications effectively for vision screening (7). SightConnect App distinguishes from other digital vision testing tools by providing patient-centric functions, including automated distance calibration for vision tests, allows to report eye-related symptoms, anterior eye photography, teleconsultation services, a referral system and assistance in locating nearby eye clinics. These allow users to access eye care screening services from home and connect with eye care providers when needed.

Patient related barriers like limited access and cost contribute to delays in care. The majority of these are conditions that are preventable but lead to visual impairment (2). In line with the World Health Organizations SPEC 2030 goals to expand access to refractive services (8) and prevent burden of common eye diseases, like cataract, corneal infections, glaucoma, diabetic retinopathy, and macular degeneration, these objectives can be achieved by eliminating the reported barriers in seeking eye care services on time and bridging the gap between the patient and providers (9). SightConnect was developed as a mobile application that can be easily downloaded in any smartphone for vision screening with integrated triage system to facilitate primary care services digitally. It is designed for use by patients and primary care providers to support timely referrals. This present study aims to validate the visual acuity testing module of the SightConnect Mobile Application compared to standard vision testing methodology in the eye clinic and evaluate the performance of its visual acuity severity-based referral categorization system.

## 2 Materials and methods

### 2.1 Study design, study settings and participants

This cross-sectional study was conducted at the outpatient eye care clinic network of the LV Prasad Eye Institute in India. Participants were recruited between august 2023 and November 2024. Consecutive patients aged from school going years (6 years) and onwards attending the eye clinics were invited to participate, regardless of their presenting visual status and ocular disease state. Written informed consent was obtained from all participants and in the case of children from their guardians. Participants who did not wish to take part in the study were excluded. The study protocol to design and validate the SightConnect mobile application was approved by the Institutional Review Board committee at the LV Prasad Eye Institute- Ethics Ref No. LEC-BHR_P-10-23-1112. The study procedures were conducted in accordance with the Declaration of Helsinki.

### 2.2 Clinical settings for visual acuity measurement

In the clinic setup, near vision was measured using the reduced Snellen near vision chart at preferred reading distance range of 35 to 45 centimeters, and distance vision was assessed using the COMPlog computerized ETDRS system at four meters. The reduced Snellen near vision chart and COMPlog are available in English letters, numbers, and Tumbling E formats to suit varying literacy levels during the in-clinic comprehensive eye exam of the participants. The median illuminance of the exam rooms was 240 lux, with an interquartile range of 82 to 397 lux. Participants who underwent a clinical eye examination as part of their appointment were recruited, and their best corrected visual acuity data were retrieved from the eyeSmart electronic medical record system.

### 2.3 Design of vision assessment module in sightConnect

The SightConnect app is available on both Android (version 7.0 and later) and iOS (version 15.5 and later) mobile operating platforms. The visual acuity testing environment in the app uses a controlled mobile screen brightness level set to its highest, which was recorded between 215 and 259 cd/m^2^ using the Konic Minolta LS-100 Luminance Photometer. The front camera system uses AI based facial landmark detection and mathematical modeling to estimate face to device distance (i.e., testing distance) during the vision test. One can perform the near vision testing by themselves by holding the phone in the range of the recommended reading distance for the near vision test and inputting the appropriate response. Individuals who were not comfortable using the mobile application requested assistance from the investigators to record their test responses. Additional personnel assistance when assessing the distance vision are required to hold the device at two-meters distance and record the subject response. During the near vision test, if the user face is beyond the reading distance range, i.e., 35–45 centimeters, the application deactivates the screen and doesn't allow user to go to the next line of the vision chart.

**Figure 1:**
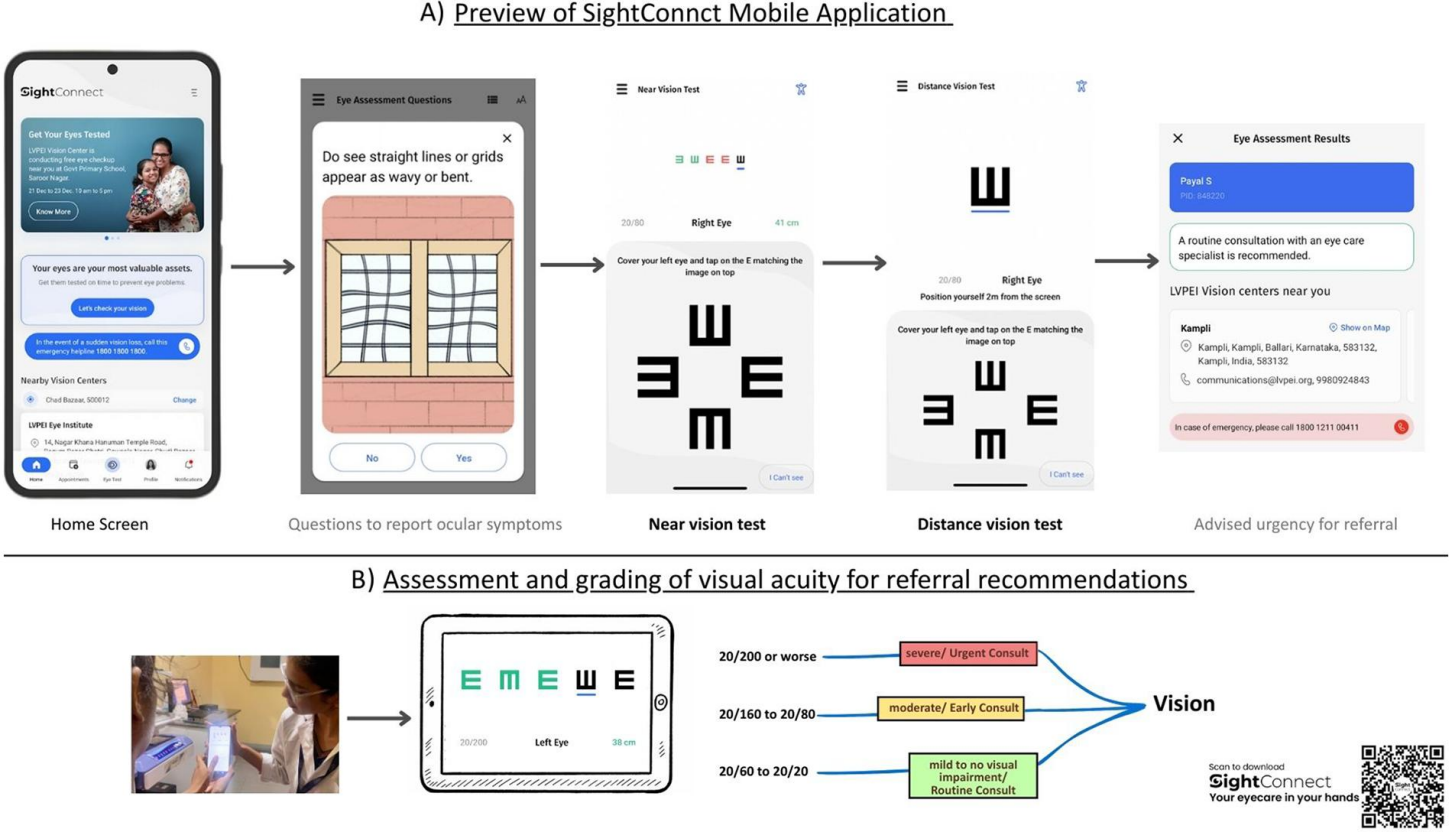
Shows the user interface and functional features of the sightConnect Mobile application.

SightConnect data were collected using Xiaomi RedmiA2 mobile phones (model 23028RN4DI, Android software version 12.0) by seven investigators in part-1 and fourteen investigators in part-2. All participants underwent a training phase to familiarize themselves with the smartphone based visual acuity testing procedure. Participants were asked to read and follow the given instructions on the screen and then train themselves on the self-testing vision exam exercise by choosing the correct orientation of the tumbling E letter. For near vision testing, participants were instructed to hold mobile phones at the acceptable reading distance during the test. Individuals who needed additional assistance to operate the SightConnect App were assisted by eye care providers. For distance acuity measurements, the examiner stood with the Smartphone at a two-meter distance. All participants were instructed to measure both distance and near visual acuity while using their habitual glasses prescription. The vision testing was performed as explained below. The SightConnect visual acuity chart uses Tumbling E letters as optotypes for both distance and near acuity measurement. The test displays five letters per line at a time for 20/200 to 20/20 acuity levels. The test begins by presenting 20/200 optotype on the screen and the test endline criteria was set for correct identification of three or more letters out of five letters on a line until the 20/20 visual acuity line. If a user response is incorrect for a presented letter, the test will return one step back and sequentially presents the previous larger letter size with five letters on a line to confirm the endline acuity score and for which direction of the three or more letters need to be identified correctly. Otherwise, the test for that given visual acuity line, distance and eye ends. At the end of the eye test the SightConnect app provides visual acuity for each eye in a Snellen fraction which is converted from the logMAR score. Additionally, the app provides details about the severity of vision to suggest referral urgency. Table 1 provides the details of logMAR acuity and categorization of visual acuity decline. For a visual demonstration of the SightConnect App features, see Supplementary Video S1 and Figure 1.

**Table 1:** Visual acuity in logMAR units and its severity based referral categorization.

Visual acuity	Visual acuity decline	Class of referral
0.0 to 0.3 (20/20 to 20/40)	No visual impairment	Routine (consultation recommended with an eye doctor yearly)
0.4 to 0.5 (20/50 to 20/63)	Mild (Grade1)	
0.6 to 0.9 (20/80 to 20/160)	Moderate (Grade 2)	Early (consultation recommended within three to six months)
≥ 1.0 (Worse or equal to 20/200)	Severe (Grade 3)	Urgent (immediate consultation recommended within 48 h)

The current version of the SightConnect mobile application allows vision testing for both distance and near visual acuity. Its in-built function classifies measured visual acuity into severity class of no visual impairment, mild, moderate and severe. Based on these grades, the SightConnect algorithm recommends referral categories by translating these terms as routine, early, and urgent recommendations for consultations (Table 1). A consensus meeting between the various ophthalmologists and vision scientists was held and agreement was sought, and the referral urgency criteria were established.

Additionally, SightConnect has symptom reporting, external eye exam, and tele-consultation appointment booking modules. A module for ophthalmic symptoms would record both visual and non-visual symptoms. These additional features were not evaluated in the present study.

## 3 Study outcomes

The primary aim of this study was to compare SightConnect visual acuity measurement with standard visual acuity measurement methods in a clinic. Our secondary objective was to evaluate the accuracy metrics for classifying the vision status into mild, moderate and severe class of visual acuity decline which forms the basis of referral as routine, early and urgent.

## 4 Statistical analysis

The data analysis was performed using Microsoft Excel and R software, version 4.5.1(R *Foundation for Statistical Computing, Vienna, Austria*). We included visual acuity data of the right and left eye in the analysis. Visual acuity assessment records from clinical evaluations and the SightConnect mobile app were retrieved from two independent cloud systems. The visual acuity data recorded in 20/20 metrics for distance vision and 6/6 metrics for near vision in the eyeSmart medical record system converted into logarithmic scale for analysis. The agreement between the SightConnect visual acuity testing and standard clinic vision testing was assessed using the Bland-Altman analysis. To account for within-subject correlations between eyes, we used a mixed effects modelling approach in the Bland-Altman agreement. Specifically, the difference between measurements (i.e., SightConnect Near Acuity-Reduced Snellen chart/SightConnect distance acuity-COMPlog) for each eye was modeled using a mixed effects model with a random intercept for patient, implemented using the *lme4* package. Model derived bias and 95% limits of agreement were calculated from variance components. The Bland-Altman plots were generated using *ggplot2* package. *tidyr* and *dplyr* packages were used for reshaping the dataset. The intraclass correlation coefficient (two-way Random, absolute agreement, average measures) was additionally performed to evaluate the reliability and consistency of the test results.

We further evaluated the visual acuity grading performance of the SightConnect app relative to standard vision testing methods. The routine, early and the urgent categories of referral urgency were compared using a 3×3 confusion matrix, which was constructed with a *caret* package. This modeling provided accuracy metrics to report sensitivity, specificity, accuracy and predictive values. A multiclass area under the curve (AUC) was calculated using the Hand-Till method. Additionally, ROC curves with one-vs-rest framework for each category of referral urgency were computed using *pROC and* visualized with *ggplot2.* The cross-tabulation summaries and accuracy metrics analysis provided in Supplementary Material 1.0, Item 2.

## 5 Results

### 5.1 Study population and participant characteristics

A total of 710 participants were recruited in part-1 and 771 in part-2. The total number of eyes that had near visual acuity was assessed was 2,962 eyes (part-1 and part-2 combined), while the distance acuity was assessed in 1,542 eyes (part 2 only). The study participants were 9 to 89 years old, with mean ages of 43 ± 20 years in part-1 and 32 ± 16 years in part-2. Gender distribution was balanced, with females comprising 45.5% in part-1 and 47.8% in part-2 term.

### 5.2 Validation of visual acuity measurements and its grading system

**Figure 2:**
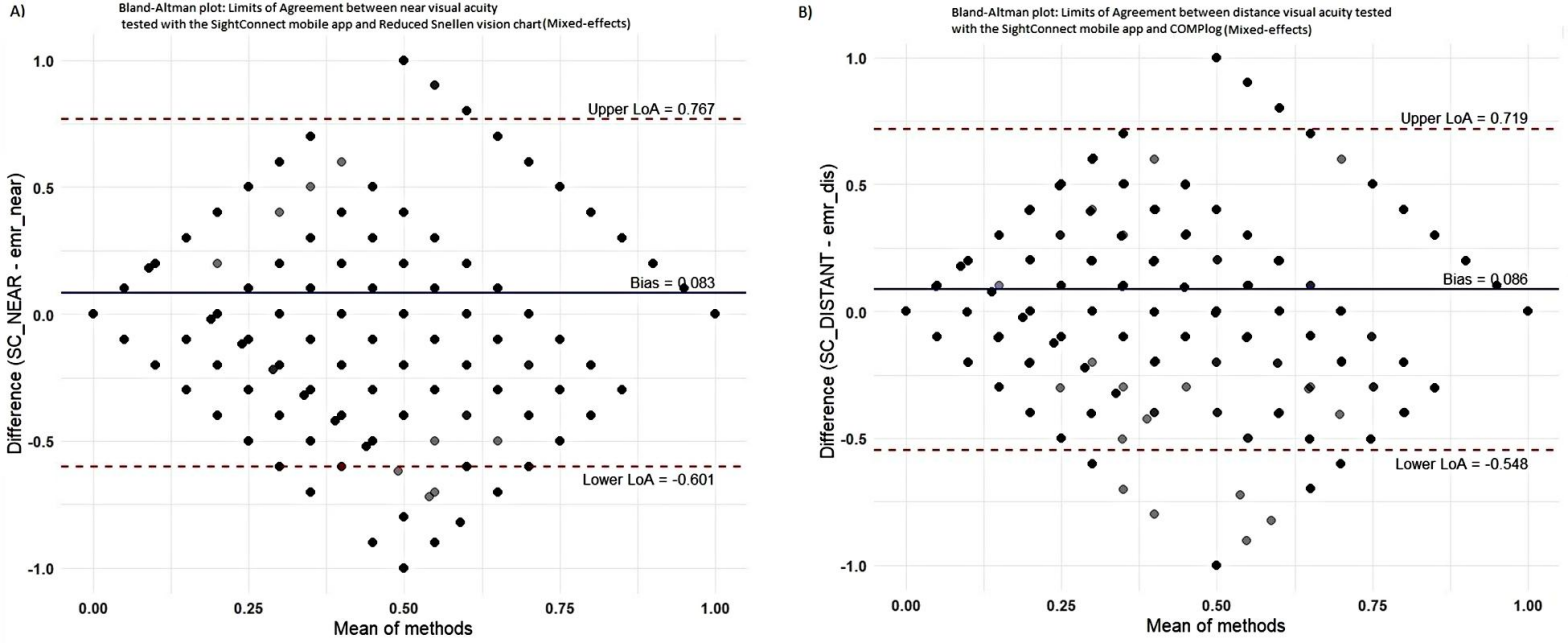
Bland-Altman plots presenting the difference in visual acuity measurements using the **(A)** reduced snellen visual acuity chart and sightConnect Mobile App at the near Reading distance, **(B)** COMPlog distance visual acuity system at 4 meters and sightConnect distance chart at 2 meters. The solid horizontal blue line in the center area represents the bias and dotted red lines represents 95% limits of agreement (LoA).

Figure 2 shown below provides the Bland and Altman plots of agreement between SightConnect visual acuity and visual acuity measured in the clinic. Table 2 provides the mean differences in visual acuity measurements obtained using acuity charts in clinic and the SightConnect App. The mean bias between the SightConnect App and standard in-clinic visual acuity measurements was four letters. Intraclass Correlation Coefficients (ICC) of 0.74 and 0.67 for near and distance visual acuity measurements, respectively, indicating good moderate reliability.

**Table 2:** Provides descriptive analysis and the strength of agreement between reduced snellen near visual acuity chart, COMPlog distance vision chart and sightConnect visual acuity measurements.

Vision assessment	N(eyes)	Mean difference, S.D.	95% Limits of Agreement (LoA)	Intraclass Correlation Coefficient (ICC)
Near Visual Acuity (Part-1 and Part-2)	2,962	0.08, 0.3	−0.60 to 0.76	0.74
Distance Visual Acuity (Part-2)	1,542	0.09, 0.3	−0.54 to 0.71	0.67

**Figure 3:**
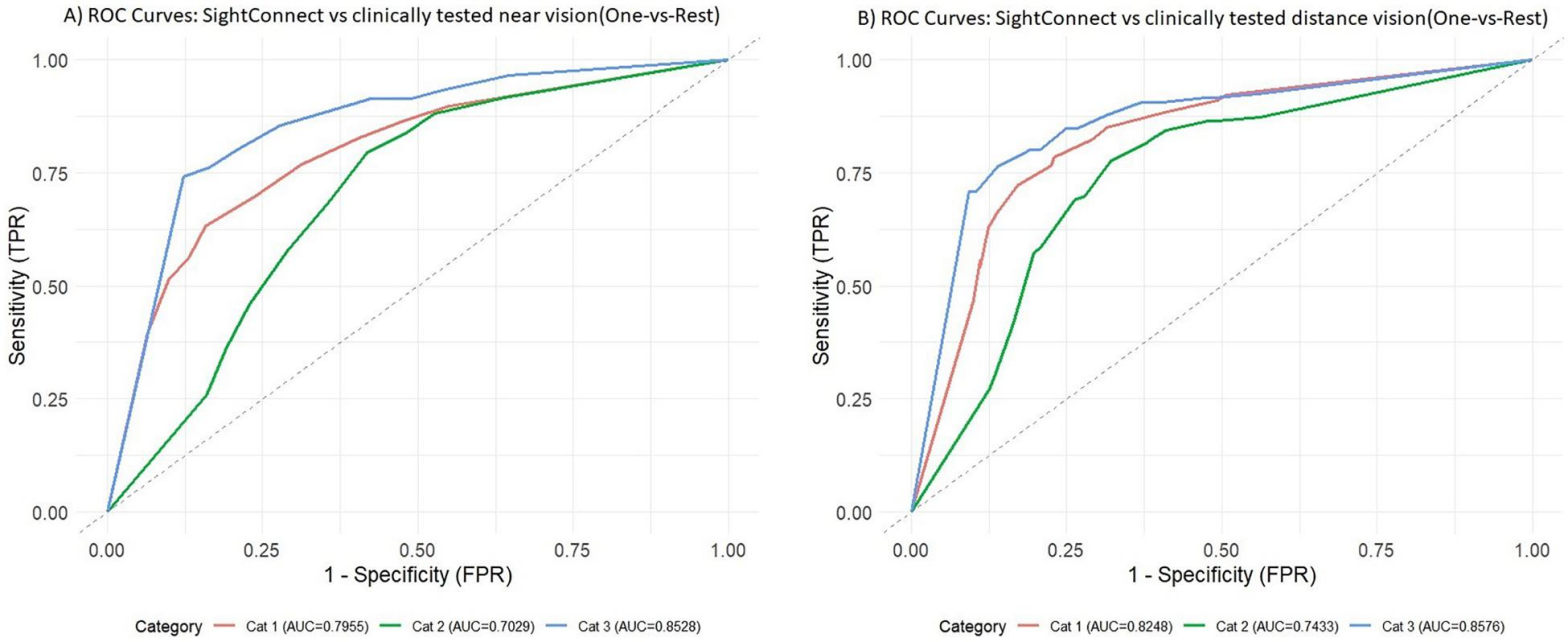
Multiclass receiver operating characteristics (ROC) curves for **(A)** near and **(B)** distance acuity-based referral categories, Cat 1(routine), Cat 2(early), Cat 3(urgent).

The ROC analysis demonstrated good discriminative performance of the SightConnect app for detecting the visual acuity decline of routine and urgent referral categories (AUC >0.8), whereas performance for the moderate visual acuity decline category was fair (AUC >0.7), see Figure 3. SightConnect visual acuity grading algorithms reported 75% accuracy for categorizing the near vision and 80% accuracy for categorizing the distance vision into no visual impairment, mild, moderate and severe vision classes for referrals, compared with in-clinic vision testing results. SightConnect App was 78 to 90% accurate for predicting urgency referrals, provided that 78.09% precise for routine, 84.23% precise for early and 86.63% precise for urgent class when predicting referral urgency using the near visual acuities. Distance vision was graded 90% accurately for urgent class, 88% for moderate visual acuity decline and 82% for routine class. However, its sensitivity and specificity for referral recommendations varied by the severity of the visual acuity decline. For near vision-based referrals, sensitivity and specificity were 0.83 and 0.59 for routine referrals, 0.2 and 0.93 for early category, and 0.74 and 0.88 for urgent category referrals. For distance vision-based referrals, sensitivity and specificity were reported 0.83 and 0.69 for routine class, 0.31 and 0.92 for early class and 0.71 and 0.91 for urgent class of referrals. Additionally, about 75% of cases were categorized as true positives in the near acuity grading model, with an underestimation of 9% and an overestimation of 17% of cases. For distance visual acuity-based referral urgency model, 80% cases were classified as true positives when underestimation observed in 5% and overestimation in 16% cases. (Supplementary Material 1.0, Item 2 provides additional information about the sensitivity, specificity and accuracy for each class of referral).

## 6 Discussion

This paper describes the validation of the visual acuity module and its grading performance to suggest referrals using the SightConnect mobile application. SightConnect mobile application demonstrated good agreement with standard visual acuity test methods, which supports its potential use for vision screening at the community and primary care levels. The Bland-Altman plot analysis showed a mean difference of 0.09 and 0.08 logMAR units for distance and near visual acuity measurements when compared with COMPlog and Reduced Snellen vision charts, respectively. These differences indicate on average a slight underestimation of visual acuity by four letters when tested using SightConnect App. From a vision screening perspective, such a difference may be advantageous and preferable for suggesting referrals as it provides a cautious estimate and minimizes the risk of missing cases that leads to severe decline in visual acuity. It also ensures cases to be categorized into a higher urgency class of referral category, aligning with the goal of early detection and intervention through vision screening followed by more referrals and reduce risk of missing pathology. Also, about 75% of cases of near acuity and 80% cases of distance acuity were graded as true positives, when compared to the standard acuity (Supplementary Material 1.0, Item 2). For each referral class accuracy may vary depending on ocular disease state (10), acknowledging that we have recruited cohorts of individuals regardless of their eye conditions and visual impairment degree for distance and near.

Notably, the comparison between the SightConnect distance acuity module and COMPlog yielded a mean difference of 4 letters. Our findings are consistent with previous validation studies of other digital vision testing tools (Table 3), where biases for testing near visual acuity ranges from −0.13 to +0.03 logMAR and limits of agreement varied depending on the digital software and reference standard used (11–14). The 95% limits of agreement between visual acuity measured by the digital visual acuity systems compared to ETDRS charts are wide in the published literature (15) and the present study. There are various factors that may account for wider limits of agreements in the present study. The sample size in the current study was the largest compared to other published studies (10). The present study included all participants both disease and healthy (16) whereas other studies included only one category of subjects with better vision (17). Further the number of investigators involved in the present study for measurement of visual acuity was 14 given the size of the population examined. Having greater number of investigators involved in the measurement of visual acuity increases variance in measurement, but such a variance is very likely to happen in real world data where multiple clinics will be involved in measurement and assessment of visual acuity. All in all, the larger limits of agreement can be explained by the reasons above and can be viewed as future challenges that require addressing. These results affirm the reliability of SightConnect in replicating standard visual acuity assessments in non-clinical settings and support its role in improving access to eye care services through validated, low-cost mobile based digital vision screening.

**Table 3:** Validation results of bias and limit of agreement reported for other digital visual acuity measurement tools compared to sightConnect.

Digital vision test software	Study (year)	Reference standard	*n*	Bias in logMAR or lines (95% LoA)
Digital software for near visual acuity test				
EyeSnellen app	Gounder et al. (13)	Reduced Snellen	122	0.001 [−0.17, 0.17]
Peek Acuity	Bastawrous et al. (11)	ETDRS	272	0.02 [−0.37, 0.41]
Peek Acuity	Bastawrous et al. (11)	Reduced Snellen	272	−0.08 [−0.50, 0.34]
Peek Acuity	Satgunam et al. (12)	Tumbling E	68	0.01 [−0.27, 0.29]
Paxos Checkup	Pathipati et al. (18)	Rosenbaum Near card	128	−0.06
V@home	Han et al. (19)	Tumbling E	163	−0.07 [−0.26, 0.19]
WHOeyes	Wu et al. (20)	Tumbling E	220	−0.02 [−0.01, 0.05]
AAPOS Vision Screening	Nik Azis et al. (21)	LEA symbols	195	0.03 [−0.19, 0.24]
Easee	Wisse et al. (14)	ETDRS	97	−0.07 [−0.52, 0.39]
Eye Chart	Ansell et al. (22)	ETDRS	24	0.02 [−0.06, 0.13]
Eye Chart	Tiraset et al. (23)	ETDRS	295	−0.01 [−0.21, 0.19]
Farsight.care Website	Bellsmith et al. (15)	Reduced Snellen Chart	146	−0.13 [−0.53, 0.27]
Verana Vision Test Mobile Phone App	Bellsmith et al. (15)	Reduced Snellen Chart	147	−0.12 [−0.50, 0.26]
SightConnect	Current study	Reduced Snellen Chart	2,962	0.08 [−0.60, 0.76]
Digital software for distance visual acuity test				
Eye Chart Pro	Zhang (24)	Tumbling E	240	0.02 [−0.14, 0.19]
DigiVis	Thirunavukarasu AJ et al. (25)	Snellen Chart	120	−0.001 [−0.18, 0.17]
Odysight	Brucker et al. (26)	4-m ETDRS	120	1.53 [−15.16, 12.11] letters
V@home	Han et al. (19)	Tumbling E	163	−0.05 [−0.25, 0.27]
University of Arizona/Banner Eye Health Chart	Bellsmith et al. (15)	Snellen Chart	147	−0.08 [−0.39, 0.25]
WHOeyes	Wu et al. (20)	Tumbling E	440	−0.05 [−0.24, 0.24]
SightConnect	Current study	COMPlog Chart	1,542	0.09[−0.54, 0.71]

Our secondary aim was to validate SightConnect's acuity grading performance for suggesting referrals. This study evaluated the performance of grading severity of vision and referral urgency using the app-specific Table 1 classification, which was developed using International Classification of Diseases 11(ICD-11) for visual acuity severity grades as a reference (27, 28). It is important to note differences between the SightConnect referral categories and ICD-11 categories. The SightConnect App considers visual acuity from 20/40 to 20/20 and 20/50 to 20/63 as appropriate for routine eye exam referral and recommends an eye doctor visit annually. The early category referral urges consultation between 3 and 6 months which uses 20/160 as upper limit. Further due to the fact that SightConnects maximum ability to measure visual acuity is limited to 20/200 (due to the screen size), an urgent referral is fixed at that point. However, ICD-11 the severe visual impairment category is defined as vision range worse than 20/200 and up to 20/400. Our consensus was that such a cutoff criterion used in the study may serve better given limitations of mobile technology screen sizes of mobile phones and the distance at which visual acuity can be measured. This may be easy to overcome with use of tablet devices which may allow the measurement of 20/400 but may limit the use of the SighthConnect mobile application as the number of individuals that have such devices in the community maybe limited. It should also be remembered that ICD-11does not have referral categories gradations to the distance and near vision acuity measurement. In our study, overestimation of referral urgency was observed in 16% of cases with distance vision grading compared to 5% of underestimation, indicating a conservative referral approach. This bias trend toward higher referral urgency (Supplementary Material 1.0, Item 2) can help reduce missed diagnoses by encouraging timely eye consultations (29, 30). However, the SightConnect algorithms currently do not collect users' medical history and previously known ocular diagnosis. This limitation may affect sensitivity for early-stage conditions such as cataract, diabetic retinopathy (DR), age-related macular degeneration (ARMD), and glaucoma—diseases that often present with progressive vision loss but may be limited by early presentation of symptoms (31). To address this issue adequately, the SightConnect platform has additional integrated triage modules that inform participants the urgency of required care and guide them for follow-up appointments with the eye care providers based on their reported symptoms, external eye photography and teleconsultations (32). Item 3 in Supplementary Material 1.0 provides a detailed description and comparison about the distinct functions of the SightConnect App compared to other digital vision screening tools. Our future goals include evaluating the combined visual acuity and symptom scores to recommend referral urgency (30). However, this present study evaluates the performance of SightConnect for vision examinations.

There are numerous reasons that can explain the differences in visual acuity estimates obtained using SightConnect and clinical evaluations. During visual acuity testing clinicians observe, guide and motivate individuals to read the optotype. This process is not necessarily addressed by portable screening tools like SightConnect as individuals may give up on first not clearly appreciated letters or not respond unless it's clear. This study did not formally look at inter examiner variability and inter- examiner reliability. All examiners were trained equally in using the SightConnect App. In this study, participants who have self-tested their vision using the SightConnect app could also have potential to introduce variability. The positioning, adherence to instructions or response reporting related bias may lead record of the false endline results. The other errors due to self-testing are possibly due to errors in reporting responses, heterogeneity in digital literacy and erroneous swiping on the mobile screen. Such participant-associated variability may lead to an error in measurement of visual acuity and may have contributed to the wider limits of agreement. The differences in luminance, viewing angle and glare due to overhead light sources may additionally account for differences in visual acuity. Perhaps asking participants to guess the optotypes at the visual acuity thresholds may decrease the difference between the visual acuity estimates of SightConnect compared to clinical evaluation. Another limitation of the study is that near vision measured by SightConnect compared with near vision assessed using the reduced Snellen chart and not with reduced logMAR near acuity chart. This may in part be responsible for variability between the two but provides a real-world comparison as most clinics use reduced Snellen in India. As SightConnect is designed for community-based vision screening (33), considering differences in literacy levels in rural and urban areas was deemed important. To account for various literacy levels tumbling E optotypes were used instead of English letters for both distance and near vision test, which may improve its utilization on large scale.

While several validated digital visual acuity tools exist (see Table 3), challenges remain in ensuring non-specialists and lay-users can reliably administer the vision screening tests, interpret results, and make appropriate referral decisions. We validated the visual acuity component of SightConnect App by lay-users utilizing digital mobile devices across various eye specialty clinics, with the assistance of a diverse group of eye care portioners, including vision technicians and optometrists. SightConnect provides access to eye care services by enabling both providers assisted and independent vision screening services. Its referral category instructions could motivate individuals to initiate conversation with eye care providers through teleconsultation services, finding local eye care services and nearby clinics.

## 7 Conclusions

In conclusion, we assessed the SightConnect mobile application's primary diagnostic functions for testing visual acuity and grading the vision. We also demonstrated the potential of vision status-based triage system in suggesting referral urgency. Furthermore, this tool can be integrated into community-based eye screening initiatives and telehealth services, offering support to both eye care and primary care providers in underserved settings.

## Data Availability

The raw data supporting the conclusions of this article will be shared by the corresponding author upon reasonable request.

## References

[1] Steinmetz JD Bourne RRA Briant PS Flaxman SR Taylor HRB Jonas JB Causes of blindness and vision impairment in 2020 and trends over 30 years, and prevalence of avoidable blindness in relation to VISION 2020: the right to sight: an analysis for the global burden of disease study. Lancet Glob Health. (2021) 9(2):e144–60. 10.1016/S2214-109X(20)30489-7 33275949 PMC7820391

[2] Alias N Buari N. Challenges and barriers to utilizing eye care services among urban population globally: a scoping review. J Health Sci Med Res. (2024):20241098. 10.31584/jhsmr.20241098

[3] Mark RG. Telemedicine system: the missing link between homes and hospitals? Mod Nurs Home. (1974) 32(2):39–42. 4493180

[4] Jagarapu J Savani RC. A brief history of telemedicine and the evolution of teleneonatology. Semin Perinatol. (2021) 45(5):151416. 10.1016/j.semperi.2021.151416 34006382

[5] Tsou BC Bressler NM. Visual acuity reporting in clinical research publications. JAMA Ophthalmol. (2017) 135(6):651–3. 10.1001/jamaophthalmol.2017.0932 28472206 PMC5847078

[6] Claessens JLJ Geuvers JR Imhof SM Wisse RPL. Digital tools for the self-assessment of visual acuity: a systematic review. Ophthalmol Ther. (2021) 10(4):715–30. 10.1007/s40123-021-00360-3 34169468 PMC8225487

[7] Davara ND Chintoju R Manchikanti N Thinley C Vaddavalli PK Rani PK Feasibility study for measuring patients’ visual acuity at home by their caregivers. Indian J Ophthalmol. (2022) 70(6):2125–30. 10.4103/ijo.IJO_3085_21 35647997 PMC9359276

[8] Keel S Mueller A. WHO SPECS 2030 - a global initiative to strengthen refractive error care. Community Eye Health. (2024) 37(122):6–7. 10.56920/cehj.785 38827975 PMC11141117

[9] Assi L Chamseddine F Ibrahim P Sabbagh H Rosman L Congdon N A global assessment of eye health and quality of life: a systematic review of systematic reviews. JAMA Ophthalmol. (2021) 139(5):526–41. 10.1001/jamaophthalmol.2021.0146 33576772 PMC7881366

[10] Thirunavukarasu AJ Hassan R Limonard A Savant SV. Accuracy and reliability of self-administered visual acuity tests: systematic review of pragmatic trials. PLoS One. (2023) 18(6):e0281847. 10.1371/journal.pone.0281847 37347757 PMC10286971

[11] Bastawrous A Rono HK Livingstone IA Weiss HA Jordan S Kuper H Development and validation of a smartphone-based visual acuity test (peek acuity) for clinical practice and community-based fieldwork. JAMA Ophthalmol. (2015) 133(8):930–7. 10.1001/jamaophthalmol.2015.1468 26022921 PMC5321502

[12] Satgunam P Thakur M Sachdeva V Reddy S Rani PK. Validation of visual acuity applications for teleophthalmology during COVID-19. Indian J Ophthalmol. (2021) 69(2):385–90. 10.4103/ijo.IJO_2333_20 33380619 PMC7933864

[13] Gounder PA Cole E Colley S Hille DM. Validation of a portable electronic visual acuity system. J Mob Technol Med. (2014) 3(2):35–9. 10.7309/jmtm.3.2.6

[14] Wisse RPL Muijzer MB Cassano F Godefrooij DA Prevoo Y Soeters N. Validation of an independent web-based tool for measuring visual acuity and refractive error (the manifest versus online refractive evaluation trial): prospective open-label noninferiority clinical trial. J Med Internet Res. (2019) 21(11):e14808. 10.2196/14808 31702560 PMC6874802

[15] Bellsmith KN Gale MJ Yang S Nguyen IB Prentiss CJ Nguyen LT Validation of home visual acuity tests for telehealth in the COVID-19 era. JAMA Ophthalmol. (2022) 140(5):465–71. 10.1001/jamaophthalmol.2022.0396 35357405 PMC8972145

[16] Hazari H Curtis R Eden K Hopman WM Irrcher I Bona MD. Validation of the visual acuity iPad app eye chart pro compared to the standard early treatment diabetic retinopathy study chart in a low-vision population. J Telemed Telecare. (2022) 28(9):680–6. 10.1177/1357633(20960640 32985378

[17] Ogino M Salmerón-Campillo RM Hunter S Hussey V Suh D Gore R Clinical validation of a novel smartphone application for measuring best corrected visual acuity. J Optom. (2023) 16(3):206–13. 10.1016/j.optom.2023.01.001 36964070 PMC10323187

[18] Pathipati AS Wood EH Lam CK Sáles CS Moshfeghi DM. Visual acuity measured with a smartphone app is more accurate than snellen testing by emergency department providers. Graefes Arch Clin Exp Ophthalmol. (2016) 254(6):1175–80. 10.1007/s00417-016-3291-4 26931323

[19] Han X Scheetz J Keel S Liao C Liu C Jiang Y Development and validation of a smartphone-based visual acuity test (vision at home). Transl Vis Sci Technol. (2019) 8(4):27. 10.1167/tvst.8.4.27 31440424 PMC6701871

[20] Wu Y Keel S Carneiro VLA Zhang S Wang W Liu C Real-world application of a smartphone-based visual acuity test (WHOeyes) with automatic distance calibration. Br J Ophthalmol. (2024) 108(11):1613–20. 10.1136/bjo-2023-324913 38514167

[21] Nik Azis NN Chew FLM Rosland SF Ramlee A Che-Hamzah J. Parents’ performance using the AAPOS vision screening app to test visual acuity in Malaysian preschoolers. J Aapos. (2019) 23(5):268.e261–e266. 10.1016/j.jaapos.2019.01.019 31520719

[22] Ansell K Maconachie G Bjerre A. Does the EyeChart app for iPhones give comparable measurements to traditional visual acuity charts? Br Ir Orthopt J. (2020) 16(1):19–24. 10.22599/bioj.146 32999989 PMC7510399

[23] Tiraset N Poonyathalang A Padungkiatsagul T Deeyai M Vichitkunakorn P Vanikieti K. Comparison of visual acuity measurement using three methods: standard ETDRS chart, near chart and a smartphone-based eye chart application. Clinical Ophthalmology. (2021) 15:859–69. 10.2147/OPTH.S304272 33664563 PMC7924116

[24] Zhang ZT Zhang SC Huang XG Liang LY. A pilot trial of the iPad tablet computer as a portable device for visual acuity testing. J Telemed Telecare. (2013) 19(1):55–9. 10.1177/1357633(12474964 23434538

[25] Thirunavukarasu AJ Mullinger D Rufus-Toye RM Farrell S Allen LE. Clinical validation of a novel web-application for remote assessment of distance visual acuity. Eye. (2022) 36(10):2057–61. 10.1038/s41433-021-01760-2 34462579 PMC8403827

[26] Brucker J Bhatia V Sahel JA Girmens JF Mohand-Saïd S. Odysight: a mobile medical application designed for remote monitoring-A prospective study comparison with standard clinical eye tests. Ophthalmol Ther. (2019) 8(3):461–76. 10.1007/s40123-019-0203-9 31346977 PMC6692804

[27] Organization, W. H. *Severe visual impairment, including blindness (ICD-11 Code 9D90)* (2019). Available online at: https://icd.who.int/browse11 (Accessed 02 January 2026)

[28] Burton MJ Ramke J Marques AP Bourne RRA Congdon N Jones I The lancet global health commission on global eye health: vision beyond 2020. Lancet Glob Health. (2021) 9(4):e489–551. 10.1016/S2214-109X(20)30488-5 33607016 PMC7966694

[29] Chan HYE Cheng JSC Bharmal A Sung VCT. The effect of visual acuity measurement on triage effectiveness in an ophthalmic emergency department. Graefes Arch for Clin Exp Ophthalmol. (2025) 263(4):1183–7. 10.1007/s00417-024-06705-5 39652183

[30] Greenwood-Lee J Jewett L Woodhouse L Marshall DA. A categorisation of problems and solutions to improve patient referrals from primary to specialty care. BMC Health Serv Res. (2018) 18(1):986. 10.1186/s12913-018-3745-y 30572898 PMC6302393

[31] Schultz NM Braunack-Mayer L Schwartz J Gaspar L. The patient experience: symptoms and impact of dry age-related macular degeneration. Ophthalmol Ther. (2021) 10(1):151–64. 10.1007/s40123-020-00325-y 33512689 PMC7886930

[32] Ahn H. Artificial intelligence method to classify ophthalmic emergency severity based on symptoms: a validation study. BMJ Open. (2020) 10(7):e037161. 10.1136/bmjopen-2020-037161 32624476 PMC7337880

[33] Marmamula S Kumar YV Rajashekar V Khanna RC. Improving access to eye care in low and middle-income countries – challenges, opportunities, and the way forward. Expert Rev Ophthalmol. (2023) 18(6):365–77. 10.1080/17469899.2023.2281448

