# Supplementary material for: Enhancing access and clinical triage in primary eye care through digital vision testing: validation of the SightConnect mobile application
Source: Front Digit Health. 2026 May 8;8:1750207. doi: 10.3389/fdgth.2026.1750207 (PMC13194495; doi:10.3389/fdgth.2026.1750207)
Supplement: Supplementary file 1 [file Datasheet1.docx]

**Item-1) Participant characteristics**

| **Participant Characteristics** | **Phase-I Period(n=710)** | **Phase-II Period(n-771)** |
| --- | --- | --- |
| Gender in percentage: Female/Male | 326(45.9%)/384(54.1%) | 361(46.8%)/410 (53.2%) |
| Age (years), mean (SD) | 38.2 ± 17.6 years | 47.2 ± 20.2 years |
| Near Visual Acuity (logMAR) | SightConnect- 0.34 ± 0.36  Reduced Snellen Chart- 0.27 ± 0.33 | |
| Distance Visual Acuity(logMAR) | SightConnect- 0.29 ± 0.35  COMPlog 0.25 ± 0.30 | |
| Education Qualification, N (in percentage)  No Schooling  School level  Graduation level  Unknown | 64(9.01%)  269(37.9%)  317 (44.6%)  60(8.45%) | 51(6.61%)  362(46.95%)  248 (32.16%)  110(14.27%) |
| Type of consultation based on economic status, N (in percentage)  Non-paying Class  General paying Class  Premium paying (Sight Saver +supporters) Class | 106(14.93%)  535 (51.55%)  69 (9.72%)69 | 462(59.92%)  303(39.30%)  6(0.78%) |

**Item 2) Cross table and classification performance metrics of urgency referrals based on near acuity results**


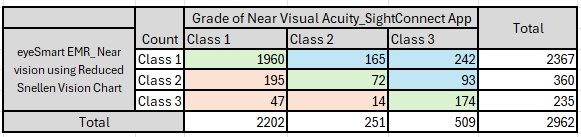


Underestimation of case category by SightConnect(n=256, *8.6%)*

Accurate case categorization by SightConnect (**n=2206, *74.5%*)**

Overestimation of case category by SightConnect (n *=*500*,16.9%)*

-------------------------------------------------------------------------------------------


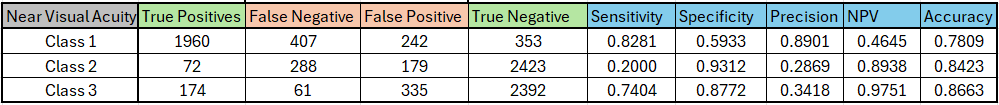


| Near vision grading | Class: 1 | Class: 2 | Class: 3 |
| --- | --- | --- | --- |
| Sensitivity | 0.8281 | 0.2 | 0.74043 |
| Specificity | 0.5933 | 0.93121 | 0.87715 |
| Pos Pred Value | 0.8901 | 0.28685 | 0.34185 |
| Neg Pred Value | 0.4645 | 0.89377 | 0.97513 |
| Prevalence | 0.7991 | 0.12154 | 0.07934 |
| Detection Rate | 0.6617 | 0.02431 | 0.05874 |
| Detection Prevalence | 0.7434 | 0.08474 | 0.17184 |
| Balanced Accuracy | 0.7809 | 0.8423 | 0.8663 |
| Overall model Accuracy | 0.7448 | | |
| 95% CI | (0.7287, 0.7604) | | |

**B) Cross table and classification performance metrics of urgency referrals using distance acuity results**


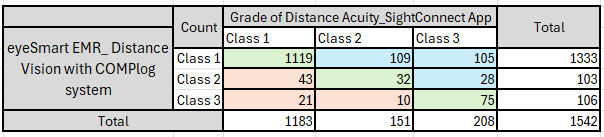


Underestimation of case category by SightConnect(n=74, *4.8%)*

Accurate case categorization by SightConnect (**n=1226, *79.5%*)**

Overestimation of case category by SightConnect (n *=*242*,15.7%)*

------------------------------------------------------------------------------------------


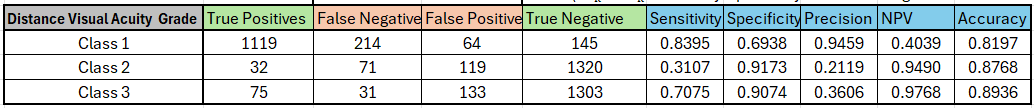


| Distance vision grading | Class: 1 | Class: 2 | Class: 3 |
| --- | --- | --- | --- |
| Sensitivity | 0.8395 | 0.31068 | 0.70755 |
| Specificity | 0.6938 | 0.9173 | 0.90738 |
| Pos Pred Value | 0.9459 | 0.21192 | 0.36058 |
| Neg Pred Value | 0.4039 | 0.94896 | 0.97676 |
| Prevalence | 0.8645 | 0.0668 | 0.06874 |
| Detection Rate | 0.7257 | 0.02075 | 0.04864 |
| Detection Prevalence | 0.7672 | 0.09792 | 0.13489 |
| Balanced Accuracy | 0.8197 | 0.8768 | 0.8936 |
| Overall model Accuracy | 0.7951 | | |
| 95% CI | (0.774, 0.815) | | |

**Item-3) Comparison of digital eye care accessibility tools- SightConnect, WHOeyes, Peek Acuity Mobile Applications**

| **Mobile Application Features** | **SightConnect** | **WHOeyes** | **Peek Acuity** |
| --- | --- | --- | --- |
| **Ophthalmologist questionnaire to report eye related symptoms** | **Yes** | **No** | **No** |
| **Near vision assessment** | **Yes** | **Yes** | **No** |
| **Distance vision assessment** | **Yes** | **Yes** | **Yes** |
| **Automated test distance calibration using AI** | **Yes** | **No** | **No** |
| **Suggests referral urgency/triage** | **Yes** | **Yes** | **No** |
| **Grades severity of vision** | **Yes** | **No** | **No** |
| **External eye photography** | **Yes** | **No** | **No** |
| **Facilitate tele-ophthalmology service** | **Yes** | **No** | **No** |
| **Locates nearby eye care providers** | **Yes** | **No** | **No** |
| **Ayushman Bharat Digital Mission compliance** | **Yes** | **No** | **No** |
| **User consent and instruction availability in Indian languages** | **Yes** | **Yes** | **No** |
| **Availability in Android devices** | **Yes** | **Yes** | **Yes** |
| **Availability in iOS devices** | **Yes** | **Yes** | **No** |

**Item-4) Visual demonstration**

See the visual demonstration of the SightConnect App features by referring the provided YouTube video link below, <https://youtu.be/7TaKogM5HOY?feature=shared>
